# The complete mitochondrial genome sequence of *Aglossa dimidiata* (Haworth, 1809) (Lepidoptera: Pyralidae)

**DOI:** 10.1080/23802359.2021.1974965

**Published:** 2021-09-17

**Authors:** Zhong-Wen Hu, Li-Kun Zhong, Chang Han, Hong-Li He, Jian-Feng Liu, Mao-Fa Yang

**Affiliations:** a Scientific Observing and Experimental Station of Crop Pest in Guiyang, Ministry of Agriculture and Rural Affairs, Institute of Entomology, Guizhou University, Guiyang, China; b Guizhou Provincial Key Laboratory for Agricultural Pest Management of the Mountainous Region, Guiyang, China; c College of Tobacco Science, Guizhou University, Guiyang, China

**Keywords:** Mitochondrial genome, *Aglossa dimidiata*, Pyralidae

## Abstract

The complete mitochondrial genome (mitogenome) of *Aglossa dimidiata* (Lepidoptera: Pyralidae) was sequenced using high-throughput sequencing. The mitogenome of *A. dimidiata* was 15,225 bp in length. It comprised 37 typical genes and one control region. All protein-coding genes (PCGs) were initiated with ATN, except for *COX1* (TTG). All PCGs used TAN as stop codon, except for *ND5* and *ND4* terminated with incomplete T. Twenty-two tRNA genes ranged from 61 to 71 bp in size. The monophyly of family Pyralidae and the sister relationship between *A. dimidiata* and *Orthopygia glanucinalis* are both supported by maximum likelihood method using the nucleotide sequences of 13 PCGs.

*Aglossa dimidiata* (Haworth, 1809) belongs to the family Pyralidae in order Lepidoptera, which is the major species producing insect tea in Guizhou, Hunan and Sichuan provinces, China (Wen et al. 2002). Insect tea is a traditional beverage made from the feces of insects feed with special plant species (Shang et al. 2013; Liu et al. 2015). Insect tea is not only rich in components such as polyphenols, flavones, caffeine, vitamin C and amino acids (Suo et al. 2016), but also is a specific resource insect product with many beneficial efficacy, such as clearing summer heat, promoting digestion, lowering blood pressure (Xu et al. 2013). Until now, none of the complete mitochondrial genome in the genus *Aglossa* has been reported. Therefore, we sequenced and annotated the mitochondrial genome of *A. dimidiata*, which will provide the useful information for phylogenetic relationships within the family Pyralidae.

The samples of *A. dimidiata* were collected from Chishui City, Guizhou Province, China (28°17′N, 105°36′E) in May 2020. The population was maintained with *Litsea coreana* in the Institute of Entomology, Guizhou University, Guiyang, Guizhou, China. The specimen and its DNA are deposited in the laboratory of the Institute of Entomology, Guizhou University, and the sample number is GUGC-Agl-00101 (Jian-Feng Liu, jianfengliu25@126.com). Total DNA was extracted from the entire body of *A. dimidiata* larvae with DNeasy Blood and Tissue kit (Qiagen, Hilden, Germany) according to the manufacture instruction. An Illumina TruSeq library was constructed for 150 bp paired-end reads and sequenced using Illumina NovaSeq 6000 platform at Berry Genomics (Beijing, China). Then, raw reads were assembled by NOVOPlasty v2.7.2 (Dierckxsens et al. 2017) and annotated by Mitoz v2.4 (Meng et al. 2019) with reference sequence from *Pyralis farinalis* (accession number: NC_047303) (Mao et al. 2019). Annotation errors were emended using Geneious Prime v2020.2.4 (https://www.geneious.com/). The annotated sequence was submitted to GenBank with accession number MW542312.

The circular mitogenome of *A. dimidiata* was 15,225 bp in size, containing 13 protein-coding genes (PCGs), 22 tRNA genes, two rRNA genes and one control region. The A + T content of the mitochondrial genome was 79.1% which was significantly biased toward AT. The total length of 13 PCGs was 10,788 bp, which was used for encoding 3,584 amino acids. And all PCGs initiated with ATN codon, except for *COXI* (TTG). All PCGs used the TAA as stop codon, except for *ND5* and *ND4* terminated with incomplete T. Twenty-two tRNA genes ranged from 61 to 71 bp in size. The length of *lrRNA* and *srRNA* were 1,388 and 814 bp, respectively.

In order to reconstruct the phylogenetic tree, we used nucleotide sequences of 13 PCGs of *A. dimidiata* and 17 reference mitochondrial genomes. Sequence of *Sesamia inferens* (Family Noctuidae) was selected as an outgroup. The maximum likelihood (ML) method was used in IQ-TREE 1.6.5 (Trifinopoulos et al. 2016) under the best-fit GTR + I + G model. The results supported the monophyly of the family Pyralidae. Meanwhile, the sister relationship between *A. dimidiata* and *Orthopygia glanucinalis* (Figure 1) was also shown. This study can contribute for future classification and evolutionary studies of the family Pyralidae.

**Figure 1.**
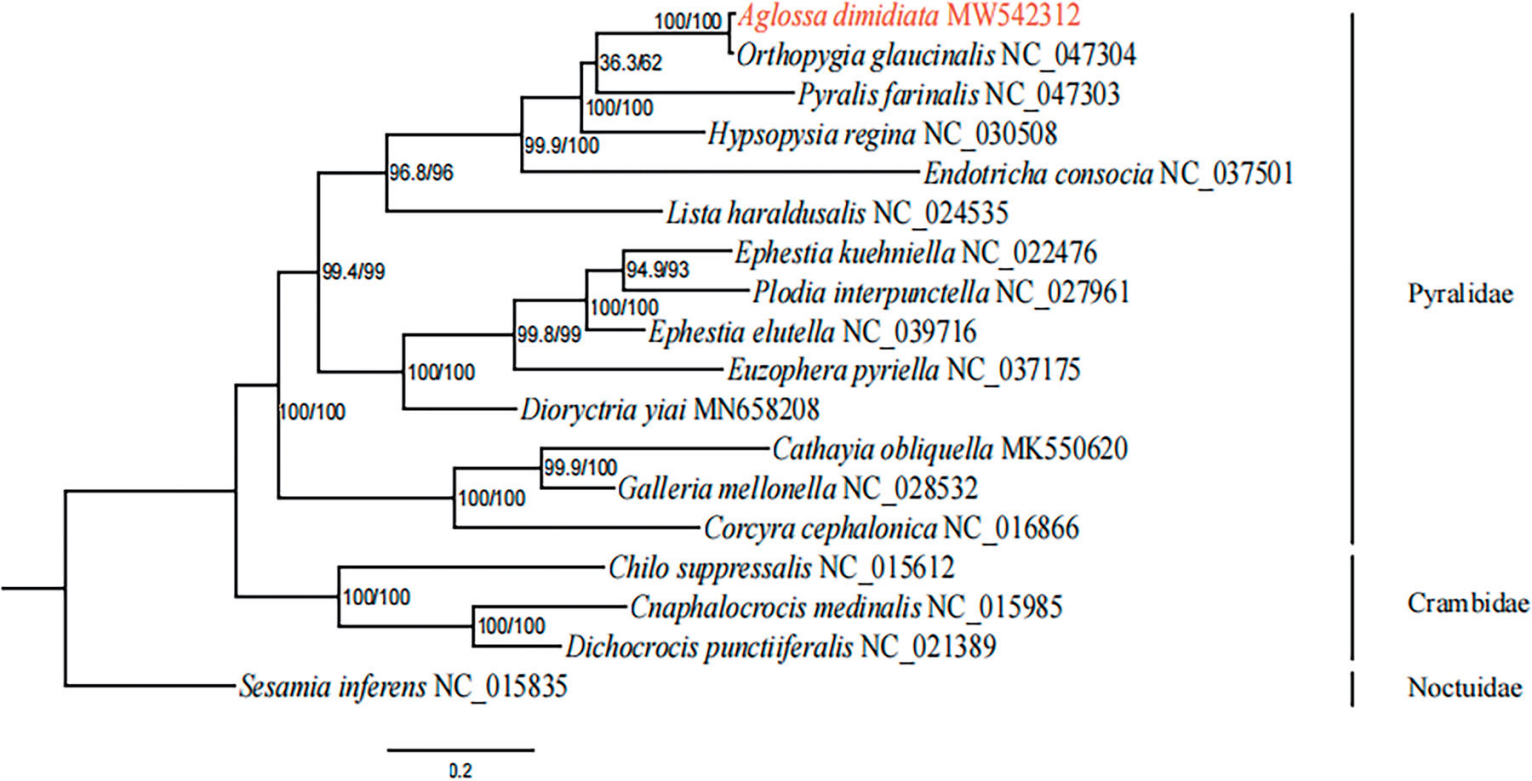
Maximum likelihood (ML) tree based on nucleotide sequences of 13 PCGs from 18 complete mitochondrial genomes with Sesamia inferens as an outgroup. Aglossa dimidiata was highlighted in red. Number at nodes represent SH-aLRT support (%)/ultrafast bootstrap support (%) (1000 replicates).

## Data Availability

The genome sequence data that support the findings of this study are openly available in GenBank of NCBI at [https://www.ncbi.nlm.nih.gov] under the accession no. MW542312. The associated **BioProject**, **SRA**, and **Bio-Sample** numbers are PRJNA707251, SRR14675281 and SAMN18806165, respectively.
